# The Role of POCUS and Monitoring Systems during Emergency Pericardial Effusion in the NICU

**DOI:** 10.3390/life14091104

**Published:** 2024-09-02

**Authors:** Cătălin Cîrstoveanu, Alexandra Bratu, Cristina Filip, Mihaela Bizubac

**Affiliations:** 1 Department of Neonatal Intensive Care, “Carol Davila” University of Medicine and Pharmacy, 020021 Bucharest, Romania; catalin.cirstoveanu@umfcd.ro (C.C.); cristina.filip@umfcd.ro (C.F.); ana-mihaela.bizubac@umfcd.ro (M.B.); 2 Neonatal Intensive Care Unit, “M.S. Curie” Emergency Clinical Hospital for Children, Constantin Brâncoveanu Boulevard, No. 20, 4th District, 041451 Bucharest, Romania; 3 Department of Pediatrics, Pediatric Cardiology, “Carol Davila” University of Medicine and Pharmacy, 020021 Bucharest, Romania

**Keywords:** PEff (pericardial effusion), cardiac tamponade, neonatal, CVC (central venous catheters), cardiac POCUS, monitoring

## Abstract

Central venous catheterization is, now, one of the most routinely used procedures in the NICUs, helping during the care of very sick infants. Pericardial effusion is a very rare but severe complication, with a high mortality. The cases described are part of an ongoing retrospective study where the use of central catheters inserted in our surgical NICU, and its complications is being analyzed. 16 cases over 13 years are presented in this article, varying in severity from mild, self-resolving cases that were discovered during routine cardiac POCUS to cases with important hemodynamic impact associated with cardiac tamponade and cardiac arrest. Due to immediate intervention, only one of the cases led to catheter-related mortality and that was under particular conditions. Our aim is to highlight the severity of this complication, the importance of early intervention, and the impact of a highly technologized unit and widely available cardiac POCUS.

## 1. Introduction

Central venous catheterization is, at the moment, one of the most routinely used procedures in the NICUs helping in the care of very sick infants, and has an essential role in the diagnosis and therapeutic process in the NICU [1].

A central catheter is defined by the position of its tip at the level of the superior vena cava (SVC), inferior vena cava (IVC), or inside the right atrium [2]. For femoral insertion, two positions are acceptable: a high position, right above the diaphragm, close to the right atrium; or a low position, in the lower IVC, downstream of the renal veins or in the iliac vein. The latter is often preferred due to the lower rate of complications [3,4]. The ideal position of the tip remains a topic of ongoing debate. While studies in adult patients suggest that an intracardiac placement of the tip does not increase the risk of complications, current recommendations for the neonatal population strongly advise against this due to higher rates of vessel erosion and cardiac tamponade. Instead, the superior or inferior cavo-atrial junction is suggested as the ideal position [5,6].

Various methods can be used to confirm the tip position, including chest X-rays, trans-thoracic ultrasonography (US), trans-esophageal US, and intracavitary ECG [7]. Although chest X-ray has been considered the “gold standard”, it has possible disadvantages such as exposure to radiation, cost, and controversies regarding the accuracy of radiological landmarks used to identify the correct tip position [7,8,9]. For catheters placed in the upper part of the body, the main radiological landmarks used to determine if the tip is inside the SVC or the atrium is the carina. While it is generally accepted that if the tip is above the carina it is located in the SVC [9,10] some argue that in pediatric populations this is not the case [8].

In our clinic, following the recommendation from the Toronto Centre for Neonatal Health in “Umbilical Catheters and Peripherally Inserted Central Catheters” [11] and Cincinnati Children’s “upper limb PICC Tip Target Position” [12] and “Lower Limb Tip Target Position” [13] the ideal tip position is consider to be outside the right atrium [11,12,13,14]. The radiological landmark used to identify this position on X-ray for SVC is a triangle bordered by the carina, T4 and T5 vertebrae, and the right main bronchus. Catheters positioned below T5 are considered to be inside the right atrium. For IVC, the ideal tip position is between T9-T11 vertebrae or at a low position. 

The use of POCUS allows for real-time tip navigation and localization during the CVC placement. First of all, POCUS allows a quick assessment of the venous system in order to choose the optimal insertion site, visualizes the needle during the venipuncture, and tracks the catheter itself inside the veins until it reaches the target position. A saline flush can help to better identify the tip [15,16]. This method is standard procedure in our unit. 

The ECG method uses the catheter itself as an intracavitary electrode, detecting the changes in P-wave as the CVC approaches the cavo-atrial junction [7]. This method allows real-time verification and has been proven to be safe, accurate, and feasible in neonates [17,18,19,20]. Despite its advantages, this method also presents limitations when very small-caliber catheters are used in emergency situations and it cannot be used for “low” femoral CVCs [16]. This method is not used by our clinic.

In neonatal patients, the volume of the pericardium is usually less than 5 milliliters; pericardial effusion is defined as the accumulation of fluid between the two pericardial layers exceeding this amount [21]. Cardiac tamponade occurs when diastolic feeling, usually of the right heart, is impaired due to the cardiac chambers being compressed by the intrapericardial pressure [22,23,24]. Cardiac tamponade is influenced not only by the volume of fluid but also by the rate at which it accumulates. Very small volumes can cause tamponade if the fluid accumulates rapidly and larger volumes can be tolerated if the accumulation occurs in time because it allows the pericardium to stretch [23,24,25,26,27,28,29].

The atrial wall structure in newborn patients might play an important role in the incidence of this complication. For this specific population, the myocardium is thinner or even absent in some sections of the atrium, which are covered only by the epicardium and endocardium and are therefore more vulnerable to both mechanical and osmotic injury, accounting for a higher risk of PEff [30,31]. Furthermore, Jonathan G. Bensley found prematurity to be associated with an abrupt reduction in cardiomyocyte cell division, which may negatively impact the total number of cardiomyocytes, reduce cardiac functional reserve, and impair the reparative capacity of the myocardium [32].

Most neonatal PEffs are iatrogenic, with CVC being one of the main causes [21]. Although this is a rare complication with a mean incidence of around 3.8‰ (ranging from 2.2‰–6.7‰), its consequences are oftentimes life-threatening; it has a mortality rate of around 27% [30].

Cardiac POCUS performed by neonatologists provides real-time information during emergency situations and subsequently helps guide the management of the case [33]. Cardiac POCUS is crucial for detecting pericardial effusion and assessing its impact on cardiac function and it can be life-saving. It also allows for direct visualization to guide pericardiocentesis when needed [33]. A cardiac effusion can be identified in any of the main 4 cardiac views [28]. Although the definitive diagnosis of cardiac tamponade requires hemodynamic and/or clinical improvement after pericardial drainage, cardiac tamponade should be suspected when a pericardial effusion is visualized along with diastolic right ventricular collapse, systolic right atrial collapse, a plethoric IVC with minimal respiratory variation, and increased respiratory variability in atrioventricular valve inflow [28,34,35].

## 2. Materials and Methods

This article presents the 16 cases of catheter-related PEff that occurred in our surgical neonatal intensive care unit, from 2011 to the present, varying in severity from mild, self-resolving cases discovered during routine POCUS to cases with significant hemodynamic impact that led to cardiac tamponade and cardiac arrest.

These cases are part of an ongoing retrospective analytic study examining the use of CVCs and their consequences in our surgical NICU over the last 13 years, involving a total of more than 6000 catheters.

The inclusion criteria were as follows: patients who had central lines inserted during the admission in our unit, developed pericardial effusion following the insertion with proof of misplacement of the tip or suspected erosion and had no further accumulation after the removal of the catheter. In cases where pericardiocentesis was performed, laboratory findings showed the composition of the extracted fluid to be similar to that of TPN, further confirming its origin. Exclusion criteria included patients with US evidence of pericardial effusion before catheter insertion, patients presenting pericardial effusion immediately after cardiac surgery, and patients with pericardial effusion but no proof of misplacement of the catheter/no improvement after the removal of the catheter.

All patients admitted to our unit since 2011 were screened for this study. A total of 26 cases of PEff were reported in our unit. Only 16 of those were deemed to have been caused by central catheters, thus meeting the inclusion criteria for this study. The remaining cases were considered unrelated to the CVCs. 

Written informed consent from the patient’s legal guardians was obtained upon admission, as it is routine practice in our hospital. 

## 3. Results

Demographics: Out of the 16 patients included in this study, 12 were born prematurely at various gestational ages, ranging from 24 to 36 weeks. All weighed less than 3.000 g at the moment of admission and catheter placement. Among the remaining 4 cases of term-born babies, one had a collagen disorder, making him more prone to injury, two had no evident risk factors, and one case presented special circumstances that will be fully disclosed later in this paper. Therefore, 75% (12/16) of the cases occurred in preterm babies with low weights at the time of catheterization. Additionally, 11/16 (68.72%) were male and only 31.25% (5/16) were female. It is important to note that our unit is a surgical NICU and not one that focuses on the care of preterm newborns, who do not constitute the majority of our patients.

**Figure 1:**
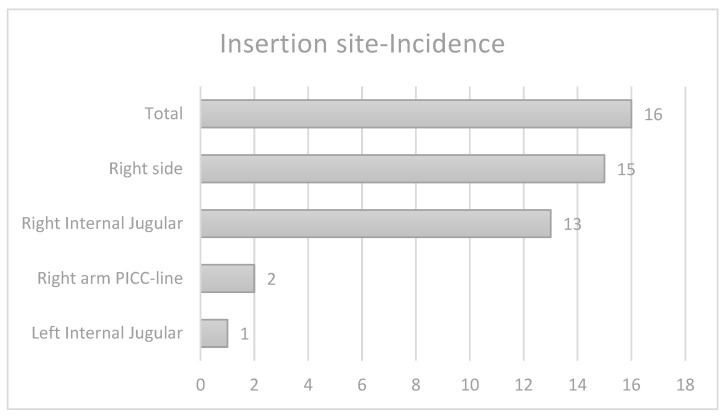
Insertion site-incidence.

Site: In 13 out of 16 cases (81%), the catheters were placed on the right jugular vein. Two were PICC lines placed on the right side (12.5%) and only one catheter that led to PEff was placed on the left side of the body, as shown in Figure 1. None of the catheters placed in the femoral veins or the lower extremities caused PEff. However, two important details should be addressed. First, the right jugular vein is the preferred site in our NICU, with the majority of the catheters being placed at this level; therefore, we cannot establish a correlation between site and incidence. Second, for the lines inserted in the lower extremity, more often than not, a low position is obtained, with the tip further from the atrium. Therefore, the position of the tip, rather than the site of insertion, may be the significant factor.

Tip position: All catheters placed in our unit are verified in multiple ways before infusion is started. First, the insertion is US-guided, and the tip position is confirmed by the ECG changes (such as modified P waves, atrial extrasystoles, or supraventricular arrhythmias) caused once it reaches the right atrium. Direct US visualization of the tip, when possible, or indirect visualization by infusing a bolus of fluid and monitoring where it exits the tip and when it reaches the atrium ensures accurate placement. Once the catheter is secured, radiological confirmation is obtained. While we, as neonatologists perform POCUS to guide catheter insertion and verify the tip’s position, preferring direct visualization of the tip, X-ray remains the “gold standard” and it is still standard procedure by our internal protocol to obtain one after the catheter is secured and have it interpreted by the radiologist.

Both direct visualization of the tip inside the right atrium by the US or its projection over the cardiac shadow on X-ray suggests that an intra-atrial position can guide clinical decisions to reposition or remove the CVC. A modified tip position on X-ray is always double-checked by POCUS before any changes are made. In some cases, a good window that permits direct visualization of the tip by POCUS is not available (for example large post-surgery dressing, modified anatomy of the heart) so X-ray is the best option.

In the past, we used to consider the catheters to be in the correct position if the tip was in the distal vena cava or inside the right atrium, though the latter is now considered contraindicated. If the catheter is inserted too deeply, it is either removed or repositioned; if it doesn’t reach deep enough, it is used as a peripheral line.

Only in 1 of the 16 cases we had radiologic findings of a deeper than preferred position from the beginning, yet the catheter was still used. The 2 catheters placed in the OR did not have radiographic confirmation of the position before infusion was started. Therefore, in 13 out of the 16 cases (81.25%) the tip position was considered to be ideal when infusion was started. The radiological findings after the PEff episode, before the catheter was removed, confirmed an intra-atrial position.

**Figure 2:**
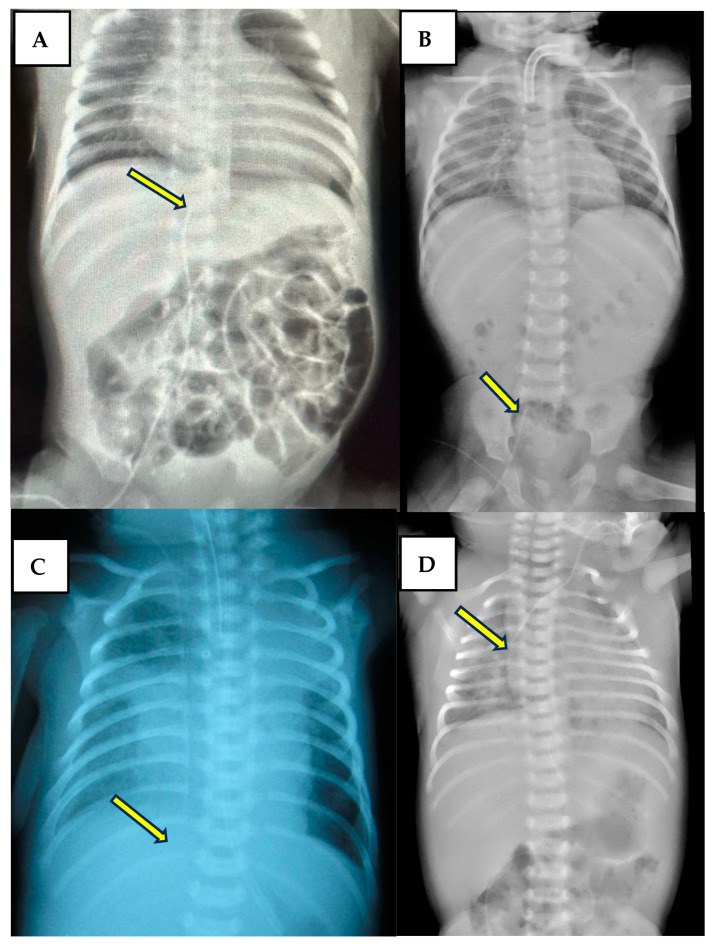
Picture (**A**,**B**)—tip placement accepted for a CVC inserted on the femoral vein (T11–T10, S1) (arrow). Picture (**C**)—CVC inserted in the upper body causing PEff due to very deep insertion (T9–T10) (arrow). Picture (**D**)—normal position of a CVC inserted in the upper body (T4) (arrow).

Radiological re-evaluation of the catheters is not done routinely in our unit, but cardiac POCUS is frequently performed, and the absence of pleural or pericardial effusion is always verified. Figure 2 shows a variety of positions of the tip.

Timing: In terms of timing, most cases occurred in the first four days after catheter insertion. For two of the cases, the exact timing is uncertain, while four occurred within hours after the placement, and all the others followed in the first 4 days. We do believe the difference in timing can be due to different mechanisms of PEff. The later-onset pericardial effusions are more likely to be related to slow erosion caused by the chemical properties of the infused substances, as we use our central lines for TPN and all medication.

Of the four cases that happened immediately, three shared a common factor we could identify, the patient had undergone surgical interventions with large volumes of fluid being infused as boluses at a fast rate and by hand, increasing the risk of catheter-related complications related to tip migration or direct mechanical damage to the vascular or cardiac wall.

**Figure 3:**
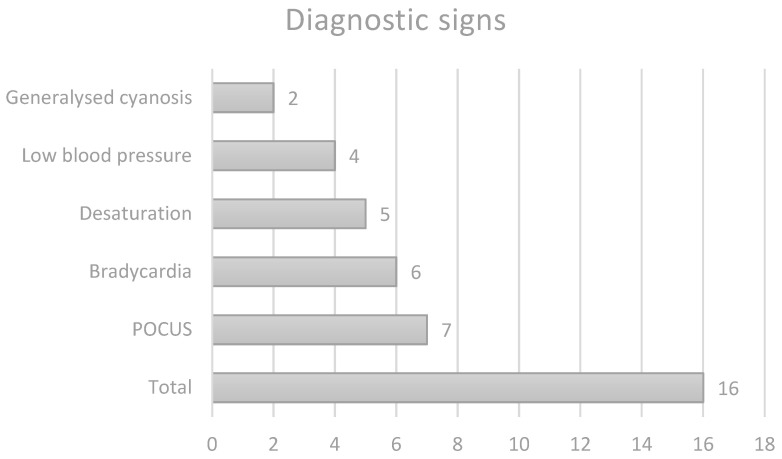
Most cases of PE were discovered during routine POCUS, before any alarm signs were noticed.

Diagnostic signs: The diagnosis of this complication was made at different stages of evolution, based on various clinical and paraclinical findings. In 4 of the 16 cases (25%) the PEff was discovered during routine cardiac POCUS, with no specific signs or concerns beforehand. In 3 cases (18.75%) PEff was discovered in the early stages, with tachycardia being the only associated sign leading to control POCUS. Therefore, 43.75% of the cases were discovered in the early phases of evolution, with minimal or no hemodynamic impact, due to the use of cardiac POCUS. In the remaining cases, the most frequently reported signs were bradycardia (6/16, 37.5%), desaturation (5/16, 31.25%), low blood pressure (4/16, 25%), and generalized cyanosis (2/16, 12.5%). A total of 9 out of 16 (56.25%) of the patients went into cardiac arrest and required resuscitation. This information is summarized in Table 1 and Figure 3.

**Figure 4:**
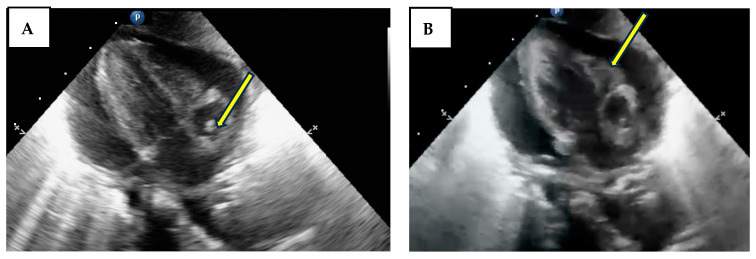
Pericardial effusion and tamponade. Picture (**A**) subcostal view-long axis-4 chambers view showing the CVC inside the right atrium (arrow). Picture (**B**) subcostal view-long axis-4 chambers view showing the diastolic collapse of the right ventricle (arrow).

For the symptomatic cases, cardiac POCUS was essential for the rapid assessment of the cause and, when needed, for guiding the pericardiocentesis performed by our team of neonatologists. Variable echocardiography findings of neonatologist US examination are shown in Figure 4.

Early intervention: Four patients (25%) needed no intervention with the situation being self-resolving. They were closely monitored with routine POCUS for any changes in the quantity of liquid and the catheters were either repositioned, removed, or used solely for blood draws. Two patients (12.5%) only needed pericardiocentesis (performed by our neonatologists at the bedside) without any need for resuscitation, both being diagnosed in the early stages, before the occurrence of bradycardia. A total of 12 cases needed pericardiocentesis (75%), 2 patients (12.5%) needed to repeat this procedure twice, and ultimately, the same 2 patients needed pericardiotomy, one after the dislodgement of the initial drain and the other because of very fast reaccumulation and suspected ventricular wall perforation. In our unit, pericardiocentesis is usually performed by our team, using a 22 G central line catheter with a Seldinger procedure most of the time, while pericardiotomy is performed by a cardiovascular surgery team in the OR (one case) or locally in the NICU (one case).

**Figure 5:**
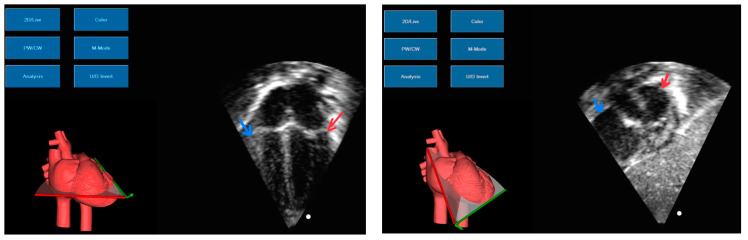
Images obtained from the cardiac US simulator. On the left—apical view, on the right—subcostal view. Red arrow showing left cavities, blue arrow showing right cavities.

**Figure 6:**
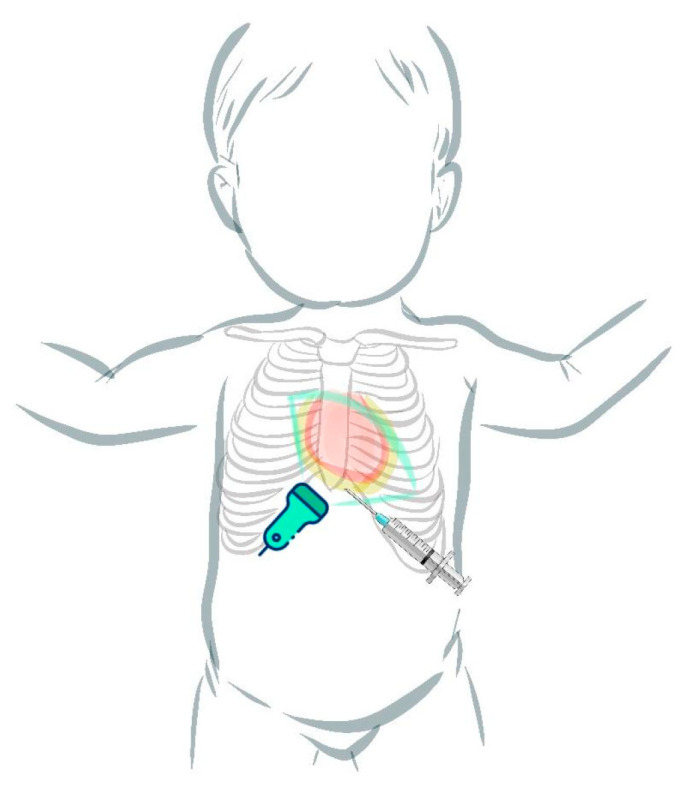
Simplification of the pericardiocentesis procedure.

Pericardiocentesis: In our unit, pericardiocentesis is performed by the neonatologists at the patient’s bedside, with the help of US guidance, shortening the waiting time for intervention. We consider this to be one of the key factors in the management of PEff in our unit. To guide the pericardiocentesis, US images are obtained in an anatomical position, to facilitate the insertion of the needle parallel to the lateral wall of the right ventricle. Two views are acquired, an apical view and a subcostal view (Figure 5). In the apical view, the needle can be seen advancing from the apex, parallel to the right ventricle. We use a short cannula for access, followed by fluid removal, insertion of a guidewire, and finally, a short polyurethane catheter insertion inside the pericardium for draining a possible reaccumulation of the fluid. A simplification of the access procedure is shown in Figure 6.

The quantity of liquid extracted was, on average, approximately 27 mL. 5/12 (41.66%) patients had 12 mL of liquid extracted, leading us to believe that this is the most common point at which important hemodynamic impact is observed in our patients. Of course, this should be analyzed in correlation with the size of the infant. Only 4 patients had more than 12 mL, specifically 15, 20 and 25 mL, respectively. Another patient had 50 mL and 60 mL at the second accumulation, but this specific case will be discussed later on in detail. This data is missing for 3 patients.

The liquid was described in most of the cases as “milky white” or “xanthochrome”. The composition of the liquid, in the cases which were analyzed, showed characteristics of parenteral nutrition, with high concentrations of lipids and glucose, indicating its origin in the fluids infused on the central line.

Later intervention and long-term outcome: Only 5 out of 16 patients needed later intervention, requiring continuous inotropic and/or vasopressor support, the longest period being 10 days of diuretics, and volume repletion. Out of the 16 cases, only one death was reported (6.25%), occurring under specific conditions that will be detailed later on. The other patients had no long-term impact of the PEff, either making a full recovery and being discharged or having a death unrelated to this complication and caused other specific ailments.

Two cases will be discussed in detail because we consider them particularly relevant for our study.

### 3.1. Case 1. Hemopericardium Due to Guidewire Perforation during CPR

A male patient, born at term from an unmonitored pregnancy was diagnosed with a diaphragmatic hernia and early-onset sepsis. From the presentation, the patient was extremely unstable and hypotensive, maintaining SpO2 no higher than 60%. His status was evaluated and the initiation of urgent ECMO therapy was decided by our team. A first CVC was placed on the left femoral vein with no complications, then the femoral arterial line. Soon after, ECMO cannulation began. The guidewire was inserted and the cannulas followed after. While the circuit was getting prepared and the cannulas were being secured, with the guidewire still inside, the patient went into cardiac arrest. Resuscitation was started immediately, with chest compressions and adrenaline boluses, with no improvement in the general state of the patient. Heart US performed by our team showed pericardial effusion in large quantities with tamponade, no cardiac activity, and the guidewire in a lower position than expected. For this reason, the guidewire was immediately removed and emergency pericardiocentesis was performed removing approximately 50 mL of blood. After the liquid was removed cardiac activity returned to a basal level. The ECMO circuit was put in place, but soon after, a new episode of bradycardia occurred. US revealed reaccumulation of blood in the pericardium and this time pleural liquid was present as well. A second pericardiocentesis was performed, extracting 60 mL of blood from the pericardium and 100 mL from the pleura. At this point, it was suspected that the guidewire perforated the ventricle and the cardio-vascular surgery team was called. They performed an emergency sternotomy and found a small tear in the ventricular wall and sutured it. Due to the sternotomy, the abdominal organs that were positioned at the thoracal level had to be reintroduced in the abdomen, so the general surgery team joined the intervention. While the ventricular defect was fixed and the herniated organs were placed back in the abdomen, the patient’s general status improved, becoming stable but with severely impaired blood coagulation function, with an INR of 7. A few hours after those events a massive bleeding episode, with fresh blood on the ETT and all drainage tubes, led to his death. While it might seem related, we consider that the pericardial effusion and ventricular wall tear were not the main factors that led to his death. Instead, neonatal sepsis with severely impaired coagulation function was the primary cause. 

### 3.2. Case 2. Alarms Paused

A post-surgery, male patient with a diaphragmatic hernia became tachycardic during the night. When the system alerted for tachycardia, multiple other alarms like “near end of infusion” or “occlusion” were also activated. A nurse entered the patient’s room to check the pumps and decided to “silence” the alarms during the nursing process. At the time, the monitors that our unit used had the “silent” and “pause all alarms” buttons located right next to each other. The “pause all alarms” function, which lasted for 3 min before needing to be pressed again to reactivate was accidentally pressed instead of the “silent” button.

During those 3 min, the patient became acutely unstable, firstly tachycardic with progressively lower blood pressure, followed by bradycardia, and ultimately, he went into cardiac arrest. Due to all alarms being paused for over 3 min, his worsening condition went unnoticed during the early stages and was discovered only later, when the patient’s state was irreversible. Cardiac US revealed massive PEff with tamponade effect. Despite all efforts, the patient couldn’t be resuscitated. After this incident, we introduced new measures with the company producing our monitors, including a “pause all alarms” function that lasts for only 1 min.

## 4. Discussion

This short series highlights the rarity and severity of this complication and the role of POCUS and our monitoring systems in its diagnosis and management.

The cases presented in this paper are part of an ongoing study analyzing the use of CVCs in our unit. The earliest case of pericardial effusion reported dates back to 2011. Since then, more than 6.000 catheters have been used, with a total of 16 cases of clear catheter-related origin and hemodynamic impact, amounting to 0.26% or 2.6‰, with only one death related to this incident.

This study has potential limitations, including the small sample size and the lack of the exact details about US findings before the PEff, as US images were not stored electronically at the time, but only on paper and they would be printed only in case of pathological findings, making it impossible for us to review them retrospectively. For these cases, the written interpretation was taken into consideration.

12 out of 16 patients (75%) were preterm babies, weighing under 3.000 g. This finding could support those of Jonathan G. Bensley, who claims that the myocardium of preterm babies has a different structure, making it more prone to injury [32].

It was observed that most of the cases happened in catheters placed using the Seldinger technique, in the internal jugular vein or in a superficial vein on the right side for PICC-lines, with only 1 case happening with a catheter on the left side and no cases for catheters placed in the lower extremities. However, it is important to mention that in our clinic, over 67% of all catheters are placed in the jugular veins, with almost 35% of the total being placed on the right side. Therefore, it is difficult to conclude a correlation between the insertion site and the risk of developing PEff.

It is important to note that, previously in our clinic, an inter-atrial position of the tip was accepted, especially for catheters inserted in the upper part of the body, with a position further from the atrium in the ones inserted in the lower body. After 5 cases were reported in a short period of time it was decided to change this practice. Based on current recommendation, an intra-atrial position is considered “too deep”.

As neonatologists, POCUS plays a major role in helping us guide catheter insertion and verify the tip’s position by direct visualization whenever possible. However, X-ray remains the “gold standard”, and it is still standard procedure according to our internal protocol to obtain one after the catheter is secured and have it interpreted by the radiologist. Based on the recommendation of the Toronto Centre for Neonatal Health in “Umbilical Catheters and Peripherally Inserted Central Catheters” and Cincinnati Children’s “Upper Limb PICC Tip Target Position”, the ideal tip position on X-ray is considered to be outside the right atrium [11,36]. The radiological landmark used to identify this position on X-ray is a triangle bordered by the carina, T4 and T5 vertebrae, and the right main bronchus. Catheters placed below T5 are considered to be inside the right atrium. In US, direct visualization of the tip inside the right atrium is considered a misplacement of the tip. Any of the two, direct visualization of the tip inside the right atrium by US or it being projected over the cardiac shadow on X-ray, suggesting intra-atrial position, can be used to guide the clinical decision. A modified position of the tip on X-ray will always be double-checked by POCUS before any changes are made. In some cases, where a good window for direct visualization by POCUS is not available (for example—large post-surgery dressing, modified anatomy of the heart, pneumothorax), X-ray remains the best.

Since the changes made in the tip positioning, only one other case of PEff was reported in our NICU.

Not all patients had direct proof of misplacement. For those who did, either direct visualization of the tip perforating the wall or a X-ray showing an abnormal location of the catheter, usually described as “too deep” was present. Resolution of the pericardial effusion after replacement/removal of the catheter confirmed the origin of the collection. If pericardiocentesis was performed, laboratory findings consistent with TPN characteristics supported the diagnosis. For the cases without direct proof, slow erosion of the vessel with fluid extravasation over time was rather suspected.

Most events occurred within the first few days after CVC insertion, consistent with other literature findings, where the median time from catheter placement to effusion onset was 3 days [30]. Different times of onset might be related to the mechanism. 

The consequences varied based on the quantity of fluid and rate of accumulation. Some cases of pericardial effusion were caught extremely early, with minimal fluid accumulation and little hemodynamic implications. They resolved with minimal or no intervention, without further impact on the overall evolution of the patient. Other cases involved large quantities of liquid, usually more than 12 mL, affecting heart diastolic function and requiring emergency life-saving intervention from our team and, sometimes, a cardiac surgery team.

It is important to mention that cardiac POCUS is performed routinely in our unit, by neonatologists, including residents, especially for patients with cardiac malformations or impaired cardiac function. This has allowed us to diagnose cases of catheter-related pericardial effusion in the early stages before any major hemodynamic implications occurred. In cases where pericardial effusion led to cardiac tamponade and impaired cardiac function, the availability of a US machine and a staff trained to perform cardiac POCUS allowed for quicker diagnosis and earlier intervention. All pericardiocentesis in our unit were performed under US guidance, with no incidents. 

Another key point in the discussion is the modern central monitoring system in place in our NICU, which allows for close monitoring of all patients simultaneously. We believe that continuous and very careful monitoring of the patient is essential to reducing the incidence of procedures required for patients in critical conditions and potentially resuscitating them or death.

**Figure 7:**
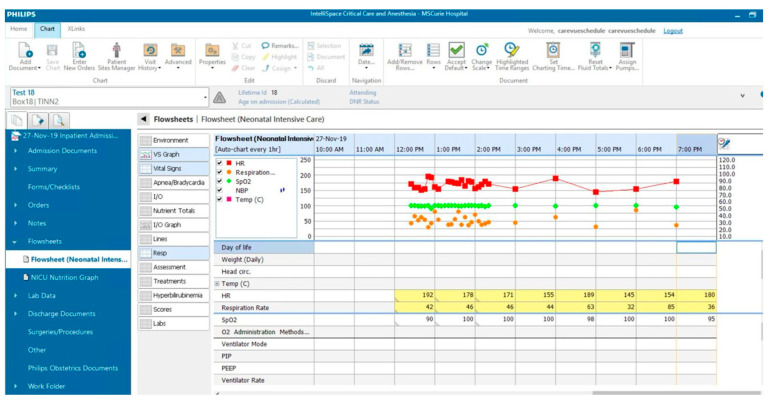
ICCA (IntelliSpace Critical Care and Anesthesia) system showing both graphic and numeric representation of vital signs.

**Figure 8:**
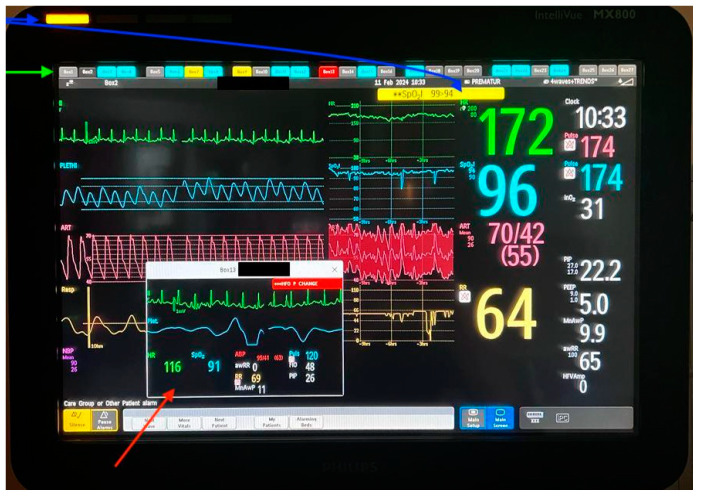
Blue arrows show there is an active alarm for this patient, consisting of a SpO2 over the limit. The green arrow shows the alarm status for all the other treatment rooms. The red arrow is a “pop-up” alarm, showing a sudden change in RR, the room number, and the patient’s name.

Each patient is placed in a single room with a monitor that collects and sends all data to the central system. This monitor displays both vitals and their trends, allowing for quick assessments. Another monitor tracks the trends of different vitals using the ICCA (IntellliSpace Critical Care and Anesthesia) system (Figure 7). Nursing needs related to the pumps (e.g., near-end of treatment, occlusion, etc.), equipment malfunction, and vitals outside the normal range trigger both visual and auditory alarms in the patient’s treatment room and outside, ensuring they are easily perceived. Important alarms from any treatment room will pop up on the screens of all the monitors so they are not missed while attending to another patient (Figure 8).

**Figure 9:**
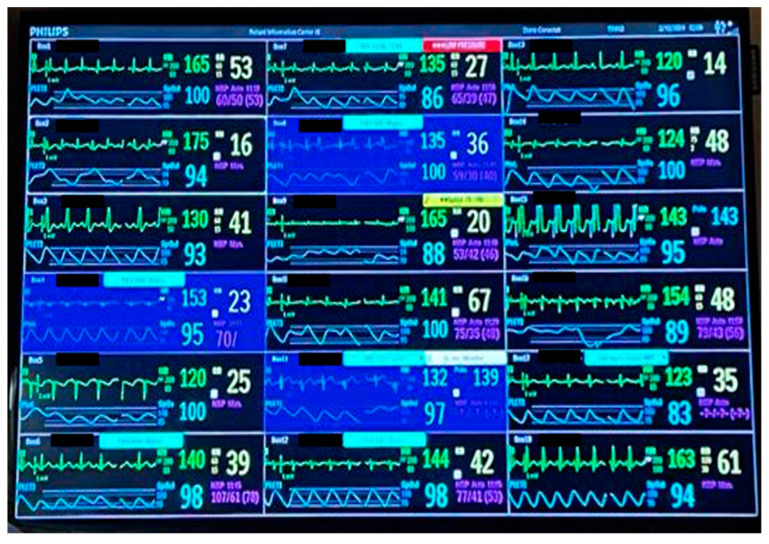
Mirroring monitors at every corner of the NICU.

Mirroring monitors showing vitals and alarms for all patients are available in each corner of the unit, being closely kept under observation by all doctors and nurses (Figure 9).

**Figure 10:**
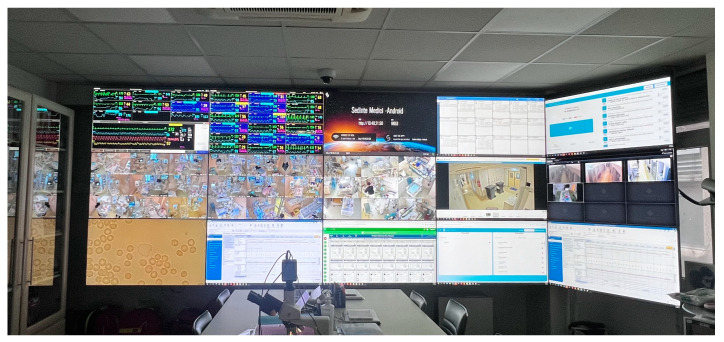
Tele-ICU room.

Inside the tele-ICU room, the central monitoring system is spread out over an entire wall, with detailed vital signs and high-resolution cameras for all patients, monitoring the whole process, allowing us to check active alarms and asses if they are real or if supplementary help is needed (Figure 10).

Our unit is connected to a telemedicine network. While we have not used it in the acute management of hydropericardium, it may be useful in departments with no staff or inexperienced personnel. Our department has the capability to perform telemedicine at any time for any patient, allowing for remote and direct visualization of the ultrasound image of the pericardium and the patient, with the guidance of a doctor experienced in such procedures. This approach helps ensure correct guidance during pericardial puncture and may also assist in placing an intrapericardial catheter until an etiological diagnosis can be made.

**Figure 11:**
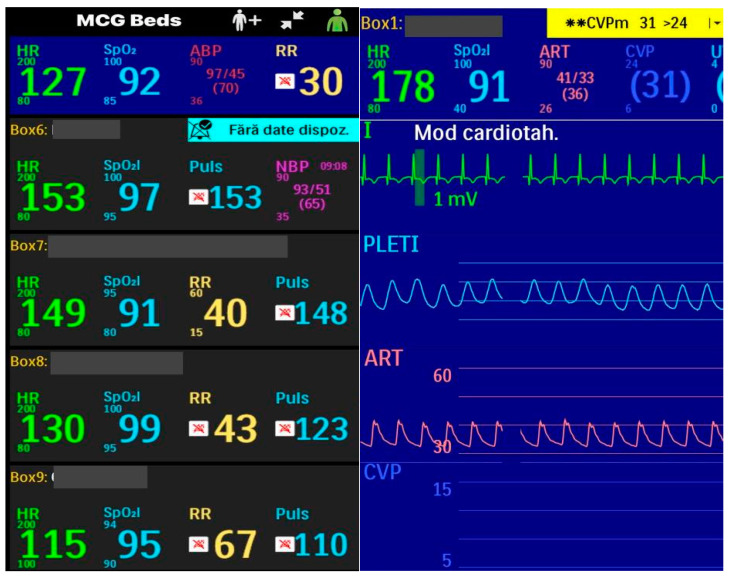
Mobile application available on every doctor’s mobile phone.

Our unit also benefits from a mobile alarm application locally and away via a VPN (virtual private network) that allows attending or on-call doctors to check the state of their patients while away from the unit temporarily, as shown in Figure 11. When patients are left “unsupervised” by doctors (only with the nurses) for a short time, using the mobile app to monitor patients from a distance can be lifesaving as it allows us to have a quicker response and minimizes miscommunications (as opposed to the nurses noticing themselves, calling the doctor and waiting for the doctor to come, the gravity of the situation not being made clear, etc.).

Two out of the 16 cases presented were caught very early on, due to the changes in heart rate trends, and 4 were caught during a routine US examination before any important hemodynamic impact was noticed. In the other cases, the complication was life-threatening and emergency pericardiocentesis had to be performed immediately. Two cases needed pericardiotomy. Even for these cases, we consider the very fast intervention thanks to the advanced monitoring and alarm system, a highly-trained team to perform cardiac POCUS, and the availability of an US machine, to be the key factors in the patients’ outcomes.

**Figure 12:**
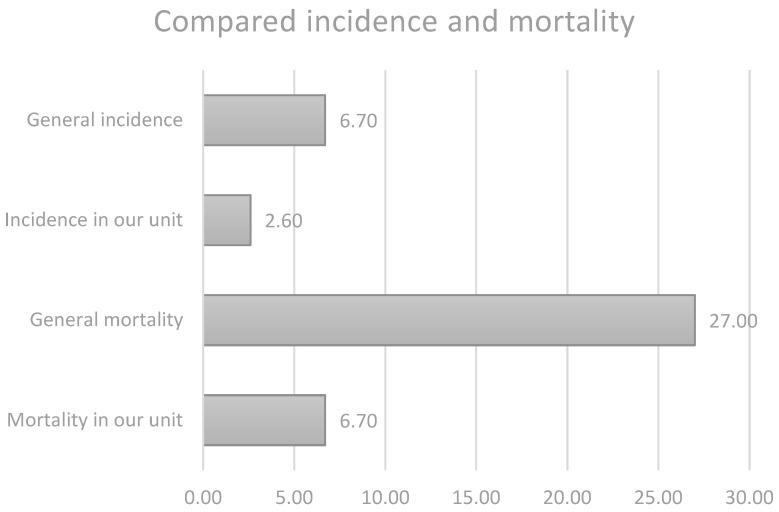
Compared incidence and mortality.

Both incidence and mortality rates were low compared to those reported in other studies (as seen in Figure 9), with only 1 (6.25%) out of the 16 patients dying because of this complication. This death occurred under unfortunate circumstances, with a failure of our monitoring and alarm system and a later-than-usual intervention, supporting the importance of this system for early diagnosis, intervention, and overall outcome (Figure 12).

The evolution of the patients after the PEff was monitored, both immediately and in the long term. While some patients needed inotropic and/or vasopressor support for up to 10 days after the PEff episode, none of the discharged patients had any long-term consequences after this episode. Some patients died later of unrelated causes due to their individual pathology and we consider that their general prognosis was not influenced by this complication.

## 5. Conclusions

The use of central venous catheters is one of the most common procedures performed in our NICU, making it crucial to continually improve protocols and minimize the risk of complications.

Although literature findings can be contrasting, we have updated our protocol over time. The optimal position for the CVC tip should be in the distal SVC or IVC rather than inside the right atrium, to prevent or significantly reduce the incidence of PEff. While radiography is still used as the “golden-standard” for confirming this position due to existing protocols, cardiac point-of-care-ultrasound (POCUS) has become a valuable tool and is often preferred, providing more and quicker information than X-rays. POCUS is not only used to guide insertion and confirm the initial tip position but also to monitor it over time. Additionally, it plays a crucial role in the diagnosis and management of PEff. 

A closer daily US observation should be adopted, especially in preterm infants with low weights at the time of catheterization, as they seem to be the most prone to develop catheter-related PEff. Special attention should be paid to the catheter’s tip position ensuring that an intra-atrial placement is avoided.

Moreover, a quick US re-verification of the catheter after the return from the OR could benefit the patient by ensuring that no dislodgement took place due to the infusion of large volumes in a short time, often administered manually.

However, the primary aim of this article is to highlight the impact of frequent cardiac POCUS performed by our neonatologists and our highly technologized unit in managing such high-emergency cases. We believe that this practice has contributed to the low mortality rates in these cases within our unit. The only death caused by PEff occurred during a system outage when no cardiac US was performed early on, further supporting our theory. While our hospital has a cardiology unit, cardiac POCUS performed as frequently as possible by our neonatologists, particularly in critical patients, provides a better chance for early diagnosis and intervention, thereby increasing the chances of survival for our patients.

This approach should help us gain a better understanding of the early, subtle changes in a patient’s hemodynamic state and provide some new insights into how to better prevent and overcome this complication.

## Figures and Tables

**Table 1:** Diagnostic signs.

	Signs on Diagnosis	Immediate Intervention	Later Intervention
P1	Restlessness, RDS, low SpO2 Cyanosis Tachycardia	Ceasing all fluid administration on CVC Monitoring	No later intervention required
P2	POCUS	Monitoring	No later intervention required
P3	Hypotension Progressive bradycardia (60–70 bpm) Cardiac arrest	Resuscitation (CPR, Adrenaline and atropine boluses) Pericardiocentesis	Continuous inotropic and diuretic support for 3 days
P4	Severe bradycardia (50 bpm) Desaturation (34%) Hypotension Cardiac arrest	Resuscitation Pericardiocentesis Pericardiotomy after pericardiocentesis cannula is dislodged	Persistent hypotension- increased doses of Dopamine Volume repletion Fluid reaccumulation
P5	Sudden onset bradycardia (60 bpm) Hypotension Low SpO2 Cardiac arrest	Resuscitation Pericardiocentesis	Persistent hypotension, metabolic acidosis- Continuous inotropic and diuretic support for days
P6	POCUS	Monitoring	No later intervention required
P7	Cardiac arrest	Resuscitation Pericardiocentesis	Persistent hypotension-Noradrenaline 24 h
P8	Bradycardia Low SpO2 Cardiac arrest	Resuscitation Pericardiocentesis	No available data
P9	POCUS-3 episodes Low SpO2- on the third episode	Monitoring	No later intervention required
P10	POCUS-initially Cardiac arrest	Resuscitation Pericardiocentesis	Inotropic and vasopressor support-10 days
P11	Bradycardia Generalized cyanosis Attenuated heart sounds Cardiac arrest	Resuscitation Pericardiocentesis	No later intervention required
P12	Bradycardia Hypotension Low SpO2 Cardiac arrest	No available data	No available data
P13	POCUS higher than basal HR (not tachycardia) Ascendent trend of the HR	Pericardiocentesis	No later intervention required
P14	Tachycardia	Pericardiocentesis	No later intervention required
P15	Cardiac arrest	Resuscitation	Exitus-nu further intervention

## Data Availability

Data are contained within the article.
